# Snakebite: Sociocultural Anthropological Bias

**DOI:** 10.1371/journal.pmed.0030412

**Published:** 2006-09-26

**Authors:** Arunachalam Kumar

While congratulating the authors of this informative article [1] for throwing light on a serious, yet much neglected health hazard, snakebite envenomation, we would like to add one more vital and cryptic cause for the abnormally high statistics in developing Asian countries: religion.

Both Nepal, cited in the paper as having the highest number of casualties, and India are predominantly populated by Hindus (in fact the only two countries in the world with Hindu majorities). In Hinduism, the cobra is, from time immemorial, revered as a vital element among the Hindu pantheon of holies. Cobra worship for countering infertility, ill fortune, or for tempering the wrath of divine curses, is not only widespread, but also firmly believed and perpetuated. India is dotted with thousands of shrines and roadside temples dedicated to the *“nag-deva”* (cobra deity).

Cobras are rarely, if ever killed when discovered in unwelcome locales; the trespassing serpents are usually trapped and released out of harm’s way [2]. The universal dread of incurring holy herpetological hexes not only allows the poisonous snake a second life, but also allows it to add its might to the ever increasing gene pool and population.

It is futile in this scenario to talk about education and awareness campaigns; thousands of years of religious indoctrination cannot be negated by education or literacy. The best, and perhaps only way, global intervention and funding can contribute to minimizing snakebite casualties is through ensuring anti-venom availability in large quantities over wide geo-locales in sub-continental Asia.
